# From division of labor to the collective behavior of social insects

**DOI:** 10.1007/s00265-015-2045-3

**Published:** 2015-12-02

**Authors:** Deborah M. Gordon

**Affiliations:** Department of Biology, Stanford University, Stanford, CA 94305-5020 USA

**Keywords:** Task allocation, Distributed algorithm, Social insects, Collective behavior

## Abstract

‘Division of labor’ is a misleading way to describe the organization of tasks in social insect colonies, because there is little evidence for persistent individual specialization in task. Instead, task allocation in social insects occurs through distributed processes whose advantages, such as resilience, differ from those of division of labor, which are mostly based on learning. The use of the phrase ‘division of labor’ persists for historical reasons, and tends to focus attention on differences among individuals in internal attributes. This focus distracts from the main questions of interest in current research, which require an understanding of how individuals interact with each other and their environments. These questions include how colony behavior is regulated, how the regulation of colony behavior develops over the lifetime of a colony, what are the sources of variation among colonies in the regulation of behavior, and how the collective regulation of colony behavior evolves.

“Such is the power of theory over rocks that it can make rocks into solid cliffs, which, however, when looked at close at hand, present openings wide enough to drive hay wagons through.” Norman Maclean (1993, p 165), in Young Men and Fire, describes his investigation of a forest fire in Montana in which many firefighters died. The official explanation for why the firefighters died had been that they could not escape because they could not run past a wall of rocks. But when MacLean went to the site, he found that there was no wall; instead, there were only scattered rocks. This led to a completely different explanation of what happened in the fire. MacLean tried to figure out why so many people held on to the wall-of-rocks version of what happened, despite all the evidence against it.

It seems to me that the history of the use of ‘division of labor’ to describe the organization of social insect colonies provides another example of the persistence of an explanation that is incompatible with the obvious facts. Leaving to others (e.g., Latour 2005) to consider why this idea has such a powerful grip on contemporary thinking, I will argue here that the field of social insect research would benefit by abandoning the phrase.

## Caste

Division of labor in social insects refers to the notion that individuals are specialized to perform particular tasks, such as foraging or cutting leaves. The phrase ‘divison of labor’ thus describes a process in which one individual repeatedly performs a task while another individual repeatedly performs another, and the phrase is often associated with an explanation for why evolution has produced that process. The core of the idea as it used in social insect research is that the causes of differences among individuals in their actions arise from differences within the individuals themselves. How an individual behaves is a consequence of some essential internal attributes, such as the sequence or activity of its DNA, the amount of certain hormones, or the size of its optic lobes or mushroom body.

When the notion of division of labor was introduced to social insect research, the emphasis was on the minority of ant genera in which adult workers differ in size, and body size was hypothesized to be associated with task. Wilson introduced ‘caste ergonomics’ in the 1960s, and in his 1971 book The Insect Societies, ideas about caste ergonomics are listed in the index under ‘divison of labor’. The original idea was that individuals are specialized to particular tasks because differences among the individuals cause particular types to perform a particular task most efficiently. For example, Wilson (1968) presents a model of the advantages of specialization in particular conditions, shown as theoretical contingency curves (“No contingency curves of actual species have yet been drawn”, p. 56). The conclusion is that specialization is advantageous: “An increase in specialization of a given caste will result, under most conceivable circumstances, in increased efficiency and colony fitness” (p. 52). The goal of the model was to predict the optimal number of castes. This optimal number was associated with the number of different body sizes, because the efficiency of performing a task depended on body size.

The notion that the advantage of division of labor was based on a link between body size and efficiency introduced some confusion from the beginning, because an individual worker’s body size persists throughout its life, but we know that an individual social insect worker’s task is not assigned for life. For example, it has been known for centuries that an individual bee moves from working inside the nest to outside the nest. This process, called ‘age polyethism’, was originally folded into the idea of individual specialization by dividing individual types into life stages; being young or old came with an assigned task. For example, Figure 1 of Wilson’s (1968) article, also in The Insect Societies (Wilson 1971, p. 342), shows three types of workers, “minor worker,” “media worker,” and “soldier,” with each one divided into temporal periods in which the ant proceeds from one task to another, nursing to defense for soldiers, and nursing to nest work to foraging for minor and media workers. The legend to the figure in Wilson (1968) specifies that each period “is treated as a separate ‘caste’” (p. 43).

By the mid 1990s, however, it was clear that flexibility in behavior goes beyond age polyethism. In both honey bees and ants, an individual’s behavior changes in response to shifting conditions and colony needs. Manipulative experiments showed that a worker’s task, such as foraging, depends on the current availability of workers to perform it (Wilson 1984; Huang et al. 1998), the current availability of materials (Jeanne 1986a), and the number of workers currently engaged in another task (Gordon 1987). This means that the task an individual performs is not due merely to the characteristics of that individual, its body size, or its age, but to a shifting set of interactions among individuals and their environments (Calabi 1988; Gordon 1989a).

I introduced the term ‘task allocation’ (e.g., Gordon 1996) to refer to the processes that determine how the colony’s effort devoted to each of various tasks, such as foraging or brood care, is adjusted to changing conditions. The task a worker performs is the result of a distributed process, influenced by interactions and changing external conditions as well as the internal properties of each individual. The goal was to shift from what philosophers call an ‘essentialist’ perspective to a ‘performative’ one (e.g., Barad 2007), to ask how does an ant come to be foraging right now rather than what makes an ant a forager. An ant is a forager when it forages, just as I am a professor when I am doing professor-like things (with the difference between an ant and me that even when not in front of a class or at the computer writing papers, I do a professor-like thing that only humans can do: I say I am a professor when asked). The reason that an ant that forages one day tends to do so the next is not that it contains some essence of forager, but that it tends to remain in the situation where the processes that make it likely to forage occur day after day, including those processes that affect its physiology, such as gene transcription and hormone levels. For example, “foraging for work” (Franks and Tofts 1994), the idea that location within the nest influences a worker’s task, is based on a performative rather than an essentialist perspective.

Oddly, task allocation, which includes the external and social, as well as internal, processes that produce the changing behavior of individuals, is now sometimes referred to as division of labor, again throwing the causal weight back inside the individuals. Suppose you want to explain why there is a traffic jam on the freeway at rush hour. Instead of trying to find out each driver’s personal story by asking, “Why are you here right now? Where did you come from and where are you going? What is your adrenaline level?” and so on, you would instead examine what are the social interactions among people, such as standard 9 to 5 work hours, that have the consequence that there are many cars on the freeway at that time. Making this distinction, between individual attributes and collective outcomes, required enormous effort in the 1980s and 1990s, with whole careers at stake. Now, 25 years later, we are still using the phrase ‘division of labor’, ignoring this long endeavor.

One confusing use of ‘division of labor’ refers to the differences between reproductives and sterile workers. Researchers studying the genetic processes associated with the differentiation of reproductives and sterile workers, picked up the phrase ‘division of labor’ in social insects and thought it meant dividing up the labor of laying eggs. (It is ironic that this use of ‘labor’ overlays so much controversy about the evolution of the reproductive physiology of social insects, in which the laying of eggs is treated as a privilege and an honor, not as work). Here, only for social insects, ‘division of labor’ is used to mean what in the rest of developmental biology is called ‘differentiation’, the process that leads a cell to take on a particular functional type. But how a larva becomes a worker or a reproductive is a process fundamentally different from those that determine a worker’s activity (including the state of being inactive) in social insects, although both are called ‘division of labor’. A worker’s activity is flexible and determined from moment to moment, while the condition of being a worker or reproductive is much less labile and involves physical transformation such as the development of functioning ovaries.

It is important to distinguish questions about morphology from those about behavior. They may be coupled, and the tightness of this coupling varies. How the relation of morphology and behavior works, and when it matters ecologically, are interesting questions. However, questions about the development of morphological features are not questions about behavior, because morphology and behavior do not map onto each other completely. Having the body of a reproductive female rather than a worker does not completely determine the insect’s behavior; it merely determines whether it is currently possible for her to lay eggs, but not whether she actually does, or if so, when. It is even more obvious that being a worker rather than a reproductive does not determine the worker’s behavior, since apparently identical workers do different things.

The equating of morphology and behavior has led to further confusion through the use of ‘caste’. First, ‘caste’ refers to lifelong social stratification in some human societies. For social insects, it has two other but contradictory meanings: both the persistent physiological differences between queens and workers, and the temporary allocation of workers into different tasks. This bifurcation in the meaning of ‘caste’ arose because in early work on ants, a worker’s task was considered to be associated with morphology, focusing on that minority of ant genera in which adult workers are of different sizes, and the sizes were the castes.

There is surprisingly scanty evidence to support the hypothesis that a worker of a given size specializes on a particular task. In fact, when the proportion of workers of each size shifts, workers switch tasks (e.g., Wilson 1984; Sempo and Detrain 2004). Even without much evidence, somehow the idea stuck that ant’s task is associated with its body size. This morphed from hypothesis to assumed fact and spread into a larger cloud that settled back onto the remaining majority of ant genera, in which ants are all about the same size, and we see ants of similar size performing different tasks. Eventually, it became clear that regardless of size, ants move from one task to another. But in a bizarre twist of nomenclature, the use of ‘caste’ continues to refer to task. This has left us talking about ‘behavioral castes’ to refer to the fluid, dynamic processes that determine which task an ant is performing at a particular time.

## Division of labor and distributed processes

The idea of division of labor is usually associated with ideas about why it helps the collective for individuals to specialize on particular tasks. The original argument, laid out in the models presented in Oster and Wilson’s 1978 book, Caste and Ecology in Social Insects, following on Wilson’s earlier models (e.g., Wilson 1968), was that the differences among individuals in morphology made a particular size uniquely best at a particular task, and that the increased effectiveness of task performance by individuals of the appropriate size increases colony fitness. This has rarely been tested in social insects, and when tested, the results have not provided strong support for this proposal. For example, Wilson (1980, Figs. 1 and 3) found that in one laboratory colony of the leafcutter ant *Atta sexdens*, ants of a range of sizes, with an average head width of 2.2 mm, cut hard vegetation. He also provided leaves to groups formed of ants of the same size and found that ants of 3.1-mm head width, which sometimes cut hard vegetation in the undisturbed colony, and those of 1.1-mm head width, which never did, both cut leaves at a rate that was about 10 (mg leaf/mg ant/min × 10^3^) lower than that of ants with 2.2-mm head width. It is not known whether the cascading and variable effects of that lower rate of leaf cutting, on the quantity of leaf fragments brought to the fungus, fungus growth, the amount of fungus fed to larvae, or numbers of ants produced, would have any influence on colony reproductive success. That would have to be tested, to support the assertion that this decrease in the rate of leaves cut per ant mass would lead to selection for colonies in which ants of 1.1-mm head width do not cut hard vegetation. If such selection pressure were demonstrated, it would still be necessary to explain why selection for an increased rate of leaf-cutting did not deter the equally slow ants of 3.1-mm head width. More generally, morphological differences in efficiency cannot explain the evolution of specialization, because the individuals performing different tasks are morphologically similar in most social insect species, including the majority of ant genera (about 276 (Blanchard and Moreau, pers. comm.) out of 326 (Bolton 2014)). It is not clear what were the evolutionary pressures on developmental trajectories (Alvarado et al. 2015) that have led, in this small minority of ant genera, to variation in body size.

By contrast, when Adam Smith (1776) introduced the idea of division of labor, the assumption was that specialization by people brings improvement because people learn through practice, and have fewer opportunities to waste time when changing tasks. He suggested that one man could learn to make candles and another could learn to make shoes, and each would get better at their trade by practicing one thing over and over, than they would if both tried to learn to do both tasks. In the same way, factory workers on the assembly line, practicing division of labor, become skilled at a particular task.

No one has ever suggested that an ant becomes a better forager because it never had to worry about learning how to take care of larvae; in fact, we know that ants usually do both. A few studies investigate whether repetition improves task performance for social insects, with mixed results, and the role of learning is not clear. For example, Dukas and Visscher (1994) found that in one honey bee colony, bees that had been foraging for 9–11 days brought in larger loads than bees that had been foraging for fewer days. By contrast, a study of 11 *Temnothorax* ant colonies showed that the extent to which an individual specializes is not correlated with its efficiency (Dornhaus 2008). In any case, for social insects, the advantage of specialization, if there were one, could not be based on learning to do only a single task in its lifetime, because workers move from one task to another.

Oster and Wilson (1978) further argued that the advantage for colonies of morphologically specialized workers came, not from learning, but from having the right numbers of each specialized type; the idea was that natural selection shapes the distribution of specialized ants into particular tasks. In an environment that favors more foraging, selection should produce colonies with a larger proportion of specialized foragers. Such selective pressure has not been demonstrated. For example, Beshers and Traniello (1996) examined the distribution of morphological types along a geographical cline that presents a gradient in the ecological conditions affecting foraging for ant colonies of *Trachymyrmex*, and found no such effect.

The advantages of a distributed process are sometimes incorrectly attributed to division of labor. Changes from one function or activity state to another, that is, the absence of permanent individual specialization, are characteristic of a distributed process. The blending of the two began with Oster and Wilson’s (1978) book, which pointed out the advantages of parallel over series processing. Parallel processing means that different parts of a task, or different tasks, can be done at the same time. If tasks are done in series, so that A must be finished before B can start, then it will take a group longer to do A and B than if both A and B are done in parallel. Individual specialization allows parallel processing, because different individuals do A and B, so the group could do the two tasks simultaneously. However, tasks can be done in parallel whenever it works for individuals to do A and B at the same time, regardless of whether they are each A and B specialists.

Oster and Wilson’s book appeared at the beginning of a revolution in computer science that led to the widespread use of distributed processing, in which similar tasks are accomplished by different parts of the system; in addition, the tasks are often run in parallel. Soon, Rumelhart and McClellan (1986) had pointed out the analogies between parallel distributed processes in computers and in brains, and these analogies have since been extended to social insect colonies many times (an early example is Gordon et al. 1992; another, although they did not refer to distributed processes, is Ratnieks and Anderson 1999). Indeed, Hofstadter used metaphorical “ants” to introduce the idea of distributed processes in Gödel Escher Bach (1979).

Distributed processes have the advantage that individual units do not have to share information about how to arrive at the collective outcome (e.g., Esponda and Gordon 2015). Instead, in a distributed process, the behavior of a unit in the system is determined by an algorithm that determines what any unit will do in a particular situation. Whether an individual component does task A or B depends on local interactions among the components, so there is no need for each individual to be informed about the state of the whole system.

Distributed processes are robust to failure precisely because the system does not use division of labor. Because there is no individual specialization, components are interchangeable and a component currently acting as one type can replace a component currently acting as another. If one unit cannot finish a task, another can take it over with an equal probability of success. This is not possible with division of labor because shoemakers have not learned to make candles.

The advantage of a distributed process goes beyond redundancy, which allows one component to replace another, identical one. The advantage of redundancy holds whether or not individuals are specialized to continue performing a certain task on the relevant timescale. If there are 20 chefs in a restaurant and only 15 are needed to make dinner, then if a few do not show up at work, there will be others to take over. But because the chefs are specialized on the day-to-day timescale, the janitor cannot do the work of a missing chef. In a distributed process, one component can replace a different one; in certain circumstances, janitors would cook and chefs would clean. Such a system might not produce a great restaurant, and indeed specialization in human societies has shaped our history in important ways (Ober 2015), but for social insects, distributed processes have proved extremely effective.

## Collective behavior in social insect colonies

The questions that we sidestep by talking about ‘caste’ and ‘division of labor’ are about collective behavior. We need to explain, not only what activity a particular worker is engaged in at any time, and what determines when that individual’s task shifts, but also how those shifts contribute to collective outcomes that allow colonies to adjust to and modify changing conditions. Such questions are central and unresolved throughout biology. It is the old contrast between nature and nurture, whether to explain phenotype, in this case what a social insect worker is doing, by what happens inside the organism or by what happens outside of it. Although everyone knows that the resolution to this is supposed to be that both matter, the challenge is to figure out exactly how.

The notion of division of labor sets aside these difficult questions. In a factory, the manager tells each worker where to go on the assembly line. In the original view of division of labor, the task of an ant depends on its body size. Because that view focused on those species in which a colony has distinct sizes of adult workers, it led to a perspective in which morphological variation was considered to be synonymous with genetic variation. The same genes, or associated genes, determined both what an ant did and what size it was. Genes controlled function, while natural selection took on the job of central control by creating the appropriate distributions of ants of each size and task.

But if we suppose that an ant’s behavior depends solely on processes inside each individual worker’s body, then how do we explain changes in its activity? In social insect biology, the explanatory power is moving over to gene regulation. Differences in the developmental trajectories that produce queens and workers, and in honeybees, transitions from working inside the nest to foraging, are associated with changes in gene expression. However, explaining what a worker does as caused by gene expression only puts the problem back one step, begging the question. First, we do not know how changes in gene expression influence behavior. In the film *Inside Out*, external events affect a person’s emotions, personified as small people living inside a girl’s brain. The emotions rush to the control panel to modify the girl’s behavior. In real life, what is the control panel and what is the machinery it controls? Are the genes the control panel, and if so, who presses the buttons, and when a button is pressed, how does the control panel change behavior? We do not know how the products of gene transcription influence a worker’s actions. We also do not know what causes gene expression to change in the first place. If the bee becomes a forager when certain genes are transcribed, rather than others, how exactly did environmental conditions stimulate the cell signaling pathways that led to shifts in the expression of transcription factors? Or does becoming a forager change its gene expression, and if so, how do we explain its transition to foraging? Of course, changing conditions, neurophysiological state, and behavior are all related, but to prioritize one over the other cuts the loop and leaves the ends dangling.

We can tackle directly the question of how worker behavior is regulated collectively, because it is possible to observe the interactions among workers and their environments that in combination with events inside their bodies produce task allocation. The collective outcome is the moment-to-moment adjustment of which workers are currently performing which tasks, and which are inactive, in response to current conditions. This is more than the assignment of particular individuals to certain tasks. The number of social insect workers that are likely to perform a particular task if they become active does not determine the number currently active performing that task. In studying social insects, we can see the processes that result in individuals changing their behavior and the processes that stimulate or inhibit activity, because we can track and manipulate those processes; for example, we can alter rates of interaction by changing the number or density of individuals present (e.g., Gordon et al. 1993; Cassill and Tschinkel 1999; O’Donnell 2001; Seid and Traniello 2006; Naug 2008).

I suggest that we abandon the phrase ‘division of labor’ and move on to new questions. ‘Task allocation’ extends division of labor to explain what each worker is doing at a given moment as a response to social interactions and external stimuli, as well as the consequence of internal characteristics of the worker. But explaining what each worker is doing takes us only part of the way to understanding the dynamics of collective behavior and when it matters ecologically. I will briefly summarize some of our studies that approach such questions, which are now being pursued by many people and have moved into the foreground of social insect research. A worker’s behavior is a symphony of interactions that connect the chemical reactions inside and among the worker’s cells, the other workers, and the rest of the world. To describe collective behavior as division of labor confines us to an obsolete perspective.

### How does a colony regulate activity?

Interactions among workers and their environments adjust colony activity to changing conditions. For example, harvester ant colonies regulate foraging activity through interactions between outgoing and returning foragers (e.g., Prabhakar et al. 2012). The rate of brief antennal contacts, in which one ant detects the cuticular hydrocarbon profile of another (Greene and Gordon 2003, 2007), sets the probability that an ant forages (Pinter-Wollman et al. 2013). An outgoing forager responds to the rate at which it meets foragers returning with food (Greene et al. 2013). Because each forager keeps searching until it finds a seed (Beverly et al. 2009), the more food is available, the more quickly foragers find it and the more rapidly they return to the nest. In this way, the rate of interaction with returning foragers is linked to food availability, so that without any global assessment, the colony can adjust foraging activity to food availability.

### How does the regulation of activity develop?

Task allocation may shift in the course of the colony life cycle. Colonies begin with founding queens and grow larger, in number of workers, as the colony grows older. It is likely that task allocation is linked to colony size in many species (reviewed in Gordon 2010). For example, the extent to which individuals remain in the same task in particular conditions changes as the colony grows older and larger, because colony size affects the probability that ants will be available to switch task in response to changing conditions (e.g., Jeanne 1986b; Gordon 1989b).

In harvester ants, nest maintenance workers are likely to switch to foraging and back in young, small colonies, but in older, larger colonies, in the absence of changing conditions from one day to the next, these two tasks are performed by distinct groups of ants (Gordon 1989b). The regulation of activity, including the proportion of the colony that remains inactive (e.g., Gordon 1992), also depends on colony age and size. For example, the behavior of older, larger colonies is more stable and more predictable from week to week, in response to perturbation, than that of younger, smaller ones (Gordon 1987). A possible explanation is that this is due to the effect of colony size on the rate of interaction among workers (e.g., Pacala et al. 1996).

### Why does one colony behave differently from another?

It is clear that social insect colonies vary in behavior (e.g., Wray et al. 2011). For example, harvester ant colonies differ in how they regulate activity using interactions (Gordon 1991; Gordon et al. 2011; Gordon 2013). Colonies live for 20–30 years, as long as the single founding queen continues to produce new workers each year. A colony’s behavior one year is consistent with its behavior in previous years, leading to persistent colony-specific trends in successive cohorts of workers (e.g., Gordon 1991). Colonies vary consistently, year after year, in foraging activity and in the way that foraging activity responds to changing conditions (Gordon et al. 2011). Consistent differences among colonies in behavior are due to behavioral reaction norms in response to conditions such as humidity (Gordon et al. 2013).

Because foraging activity is regulated by interactions between outgoing and returning foragers, differences among colonies in foraging activity must be associated with differences in how ants respond to interactions. We are currently investigating variation among colonies in the way that ants respond to interactions, adding up interactions to decide whether to leave the nest (Davidson et al. in prep). Preliminary transcriptomic analysis suggests that colony variation in the regulation of foraging is associated with neurophysiological differences in biogenic amine systems. The next step is to examine the neurophysiology of an ant’s response to interactions.

### How does the collective regulation of colony activity evolve?

Variation among colonies in the regulation of behavior is the starting point for natural selection, because colonies are the reproductive individuals of social insect populations. The ways that activity is adjusted to a changing environment create the phenotype, and natural selection acts on this phenotype (Gordon 2014). Which features of the phenotype are favored by selection is an empirical question that can be investigated using the methods of evolutionary ecology (Gordon 2011). Rather than deciding *a priori* that some measure of efficiency or colony growth is what natural selection ought to optimize, we can ask what is actually occurring in natural populations.

Using data on a population of about 300 colonies censused since 1985, we used microsatellite variation to identify colonies that were founded by the daughter queens of particular parent colonies (Ingram et al. 2013). (Here, we measured only the female component of reproductive success; each queen mates with many males and we did not track the males). Once we were able to match up parent and offspring colonies, it was possible to estimate realized colony reproductive success in numbers of offspring colonies per parent colony.

Comparing the behavior of parent and offspring colonies suggests that variation among colonies in the regulation of foraging activity is heritable from parent to offspring colony. Offspring colonies resemble parents in the regulation of foraging, in particular in the choice of days in which to reduce foraging (Gordon 2013). Founding queens disperse far from the parent colony, so parent and offspring colonies do not meet. A daughter queen apparently produces workers that respond to interactions with each other, and current weather conditions, so as to collectively regulate foraging in a way that has the same outcome as the regulation of foraging by her worker sisters (her mother’s daughters), in her parent colony.

Using this measure of reproductive success, we were able to determine empirically how natural selection is currently acting on one aspect of task allocation in harvester ants, the regulation of foraging activity. We examined how variation among colonies in the way that they regulate foraging activity, in response to changing conditions, is associated with variation among colonies in reproductive success, in numbers of offspring colonies. Colonies that forage less when it is hot and dry are more likely to have offspring colonies (Gordon 2013). A colony stores food for many months (Gordon 1993), so bringing in less food does not lead to starvation; there was no association between foraging activity and colony survival (Gordon 2013). In the severe and deepening drought of the past 15 years in the southwestern USA, natural selection is favoring the colonies in which outgoing foragers are more reserved in their response to interactions with incoming foragers, thus sacrificing food intake to conserve water. We do not know how conserving water translates into reproductive success; perhaps colonies with a larger water supply can produce better hydrated reproductives to leave on the annual mating flight.

## Conclusion

Many people are now pursuing the research questions outlined here, but some still refer to the processes they are studying as ‘division of labor’. In Alice in Wonderland, Humpty Dumpty claimed it did not matter how you decide to use a word, as long as you pay it its wages on Saturday night, but I think he was wrong. Talking about division of labor keeps pulling us back into looking for something that is not there. If we envisage a worker’s behavior as fully determined by events inside the worker’s body, it is harder to figure out how the colony’s collective behavior works and how that collective behavior evolves. To do that, we need to learn how interactions among workers and feedback from the environment combine to create the ongoing, shifting pattern of activity that determines what a colony accomplishes in a changing world.
